# Improving Mildew Resistance of Soy Meal by Nano-Ag/TiO_2_, Zinc Pyrithione and 4-Cumylphenol

**DOI:** 10.3390/polym12010169

**Published:** 2020-01-09

**Authors:** Wenping Li, Mingsong Chen, Yanchen Li, Jingmeng Sun, Yi Liu, Hongwu Guo

**Affiliations:** 1 MOE Key Laboratory of Wooden Material Science and Application, Beijing Forestry University, Beijing 100083, China; bjfu130524211@163.com (W.L.); chen_boss@bjfu.edu.cn (M.C.); lyc100083@163.com (Y.L.); tulipsjm@163.com (J.S.); 2 Beijing Key Laboratory of Wood Science and Engineering, Beijing Forestry University, Beijing 100083, China; 3 MOE Engineering Research Center of Forestry Biomass Materials and Bioenergy, Beijing Forestry University, Beijing 100083, China

**Keywords:** soy meal, antifungal, adhesive

## Abstract

As a byproduct from the soybean oil industry, soy meal can be reproduced into value-added products to replace formaldehyde as a plywood adhesive. However, the use of soy meal has been limited by its poor antifungal and antiseptic properties. In this work, three kinds of material, namely nano-Ag/TiO_2_, zinc pyrithione, and 4-cumylphenol were applied to enhance the mildew resistance of soy meal via breakdown of the cellular structure of mildew. The fungi and mold resistance, morphology, thermal properties, and mechanism of the modified soy meal were evaluated. The success of the antifungal and antiseptic properties was confirmed by Fourier transform infrared spectroscopy (FTIR) and scanning electron microscopy. The results indicated that all three kinds of material improved the fungi and mold resistance of soy meal, and sample B, which was modified with a compound of nano-Ag/TiO_2_ and zinc pyrithione, was the effective antifungal raw material for the soy-based adhesives. FTIR indicated that the great improvement of antifungal properties of soy meal modified with 4-cumylphenol might be caused by the reaction between COO– groups of soy protein. This research can help understand the effects of the chemical modification of nano-Ag/TiO_2_, zinc pyrithione, and 4-cumylphenol on soy meal, and the modified soy meal exhibits potential for utilization in the plywood adhesive industry.

## 1. Introduction

Formaldehyde-based adhesives have predominated in the plywood industry market due to their advantage with regard to cost and availability, and most of them use petrochemicals as raw material [1,2]. However, such extensive usage of petroleum, a nonrenewable resource, triggers the environmental problem of volatile organic compound release and causes human health problems [3]. Currently, the growing awareness of the need for formaldehyde-free environmental protection has motivated an intense effort to develop eco-friendly materials. In particular, renewable biomaterials such as proteins [4,5,6], carbohydrates [7,8,9], tannins [10,11], and citric acid [12,13] have been extensively studied for the replacement of petroleum-based wood adhesives.

Among these biomaterials, soy meal (SM) has attracted the interest of researchers due to its low cost, short production cycle, abundance, biocompatibility, ease of handling, low press temperatures, and the ability to bind wood with relatively high moisture content, representing a very practical material for wood adhesive [14,15,16]. However, the deficiency in fungi and mold resistance, the low water resistance, and insufficient mechanical strength of SM-based adhesive has restricted its utilization. Recently, the problems of the adhesive water resistance and properties have been basically solved with the efforts of many researchers. Zhang et al. [14] investigated a high-performance soy-based adhesive by mixing aminated soybean soluble polysaccharide (A-SSPS) with soy protein isolate (SPI) and bio-based triglycidylamine (TGA) with a hyperbranched cross-linked structure. Zhang et al. [5] achieved a remarkable boiling water resistance of soy protein-based adhesives via organosilicon–acrylate microemulsion and epoxy synergistic interfacial enhancement, which improved the adhesive shear strength to 1.20 MPa in boiling state. However, the storage time of soy-based adhesives is relatively short, and the wood-based panels pressed from them are prone to mildew and the spread of molds in the air can adversely affect human health. Soy-based adhesives have little or no mold resistance unless protected with preservatives, especially under continuous exposure to high moisture conditions by deterioration [17]. The vulnerability of SM to fungi and mold arises from its high protein and rich nutrient content, which creates a favorable environment for mold growth. 

Hence, intense efforts have been devoted to enhancing the fungi and mold resistance of SM-based adhesives. In the early days, the Forest Products Laboratory performed a mold exposure experiment on plywood panel with different water-borne solutions, and found that chlorinated phenols with a high concentration level have a good effect on mold resistance. A special glue-grade of 2-chlororthophenylphenol chlorophenols, sodium chlorophenates, orthophenylphenol, and sodium orthophenylphenate produced good results in the test on sweetgum sapwood plywood. 

As a nonmental versatile fine chemical raw material, 4-cumylphenol is an effective chemical substance applied as a fungicide, preservative, and anti-termite agent [18,19,20,21]. Higaki developed a wood preservative containing 4-cumylphenol. It was shown that 4-cumylphenol has a microbicidal reaction to mold. Resins could reinforce microbicidal activity of 4-cumylphenol and impart sustainability. Xing et al. [18] applied 4-cumylphenol to the glue line as a preservative in order to alleviate mold growth on soy-based adhesives. It was shown that the mildew resistance on the adhesive surface was better controlled when 4-cumylphenol was in the content range from 1.5 to 2.0 wt %.

Silver nanoparticles are applied broadly, resulting in non-reversible detrimental changes to bacterial cell structure, inhibition of their further growth, and cell death [22]. TiO_2_ photocatalysts have been investigated extensively for the killing or growth inhibition of bacteria [23,24,25,26,27]. Moreover, it was found that Ag metal additives may enhance charge separation as well by forming a Schottky barrier at the metal–photocatalyst interface and improve antibacterial properties [28]. Furthermore, nano-Ag/TiO_2_ composites can also act against silver-resistant microorganisms due to their photooxidative mechanism, and they have excellent antibacterial properties [29]. 

Zinc pyrithione (ZPT), a general inhibitor of membrane transport processes in fungi and cell division in bacteria, has been extensively used in soap and non-soap synthetic detergent compositions, and other products where an antibacterial action is desired [30]. In the early years, Kooistra [31] demonstrated that ZPT possesses antibacterial and antifungal properties as well as a lasting antibacterial and antifungal effect. Wei et al. [3] found that ZPT can be applied to a cosmetic personal cleansing composition at a ratio of at least about 0.01% of the weight of the composition due to its fungistatic and bacteriostatic properties.

In this research, we chose three kinds of mildew-proof agents to add into SM in order to test their anti-mold capacities. SM is a byproduct of the soybean oil industry, and the utilization of SM can add value to agricultural byproducts and decrease waste generation. The purpose of the study was to develop a new high mildew-proof modified SM which could be used to prepare an SM-based plywood adhesive for expanding the application range of plywood and extending the life of plywood.

## 2. Materials and Methods

### 2.1. Materials

SM with 43.5% soy protein content was obtained from the Huifu and Oil Company of Hebei province, China. Nano-Ag/TiO_2_ was made using a sol–gel process with 2% Ag. ZPT was obtained from SR Japanese Shoji Co., Ltd. 4-Cumylphenol was obtained from Beijing Li Xing Gong Mao Co., Ltd. 

### 2.2. Adhesive Preparation

The different modified SMs were prepared as follows (Table 1).

For SM (K): SM (28 g) was mixed with water (70 g) and stirred for 5 min at 25 °C using a stirrer with 1500 r/min.

For SM/nano-Ag/TiO_2_ for 4 samples (A1–A4): Nano-Ag/TiO_2_ (0.5, 1, 2, 3 g) was added into SM and stirred for 5 min at 25 °C using a stirrer at 1500 r/min.

For SM/nano-Ag/TiO_2_/ZPT for 4 samples (B1–B4): Nano-Ag/TiO_2_ (0.25, 0.5, 1, 1.5 g) and ZPT (0.25, 0.5, 1, 1.5 g) were added into SM and stirred for 5 min at 25 °C using a stirrer at 1500 r/min.

For SM/4-cumylphenol for 4 samples (C1–C4): 4-Cumylphenol (0.5, 1, 2, 3 g) was added into SM and stirred for 5 min at 25 °C using a stirrer at 1500 r/min.

### 2.3. Observation of Preservative-Treated SM 

Prepared samples were put into culture dishes at 28 °C and 80% humidity using a temperature humidity chamber for 15 days. Samples were observed on days 0, 1, 2, 3, 4, 5, 6, 7, and 15, and a photo was taken for records. On the first, fifteenth, and thirtieth day, 4–5 g were taken from all types of samples for detection (FTIR, TG, SEM, solid content).

### 2.4. Solid Content

The procedure was also adapted from Li et al. [32]. Three parallel groups (3 g, weight α) were dried in an oven at 105 ± 2 °C for 2 h, then weighed and recorded as weight β. The solid content of the adhesive was calculated via Equation (1). The average of three parallel groups was calculated to obtain the final value.

(1) $$Solid\;content=\frac{Weight\beta }{Weight\beta }\times 100\%$$

### 2.5. Fourier Transform Infrared (FTIR) Spectroscopy

The procedure was adapted from a previous study by Lin et al. [33]. The sample was placed into a 120 ± 2 °C oven for 2 h to cure completely, after which the cured sample was ground into a 200 mesh powder. The powder was first mixed with KBr crystals at a ratio of 1/70 and then pressed to form an adhesive folium. The FTIR spectra were then recorded on a Nicolet 7600 spectrometer (Nicolet Instrument Corp., Madison, WI, USA) from 500 to 4000 cm^−1^ with a 4 cm^−1^ resolution using 32-times scans.

### 2.6. Thermogravimetry (TG)

The procedure was adapted from one previously reported by Zhao et al. [5]. The cured adhesive powder (200 mesh) was prepared in the same manner as for the FTIR measurement. The weight loss of the sample was recorded using a TG instrument (TA Q50, Waters Corp., Wood Dale, IL, USA). About 5 mg of the powder was scanned from 30 to 600 °C at a heat rate of 10 °C/min under a constant nitrogen atmosphere (60 mL/min).

### 2.7. Scanning Electron Microscopy (SEM)

The procedure was adapted from the one reported by She et al. [34]. The sample was cured in an oven at 120 ± 2 °C for 2 h, after which the cured sample was cracked into small pieces. Several pieces of cured sample were placed into a desiccator for 2 days prior to testing. The surface of the sample piece was sputter-coated with gold before observation under a Hitachi S-3400N scanning electron microscope (Hitachi Science System, Ibaraki, Japan).

## 3. Results and Discussion

### 3.1. Evaluation of Mold Resistance for Preservative-Treated SM

Visual Observation of the Sample Surfaces

All the samples showed mold growth during 15 days at 28 °C and 95% RH (Table 2). Off-white colonies were first detected on the untreated SM (sample K) rather than on the preservative-treated ones, which suggested that the untreated SM is more vulnerable to microbial attack than the preserved ones.

On the first day, sample K presented small spots of white colonies and the surface of the sample became a little bit uneven. Colonies were not observed for the other samples.

On the second day, a large number of visible off-white colonies suddenly appeared on samples A1 and A2, which contained 0.5% and 1% preservative A, respectively. At the same time, microorganism damage also appeared on sample A3, and the color of the colonies was pink and white in interval distribution. Meanwhile, some white colonies occurred on sample C1. The surfaces of samples B1 to B4 and samples C2 to C4 retained their original appearance.

On the fourth day, samples A4 and C2 were contaminated by colonies. While the infecting process of sample C2 was moderate, the surface of sample A4 was fully covered with colonies and changed to mycelium color until the 15th day.

**Figure 1:**
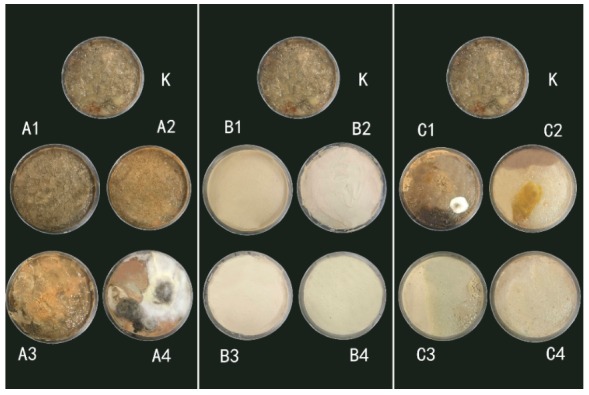
All soy meal samples were conditioned at 28 °C and 95% RH for 15 days.

After 15 days of observing time, samples B1 to B4, which contained preservative B content of 0.5%, 1%, 3%, and 5%, respectively, had no observable infected area. This phenomenon could also be viewed in samples C3 and C4, which contained preservative C content of 3% and 5%, respectively. These facts show this dose of mildew is effective (Figure 1).

Throughout the experiment, sample K was contaminated by colonies rapidly. This was to be expected, as the unprotected soybean adhesive can be expected to deteriorate rapidly from molds and possibly from other microorganisms [17]. The research of Xing et al. also confirms the soybean adhesive’s characteristic of easily growing mildew [18]. Samples B1 to B4 showed excellent antimicrobial properties during the experiment. The surface of the samples was visually observed to not be mildewed. This is due to the compounding system of the antifungal agent of nano-Ag/TiO_2_ together with ZPT. 

Samples C1 to C4 contained the preservative 4-cumylphenol. As the concentration of the mold inhibitor increased, the ability to inhibit mold growth was increased, and when the amount added reached 2%, the sample surface was visually observed on the 15th day to still be as it was originally. We attribute this to the antibacterial effect on phenolic hydroxyl functional groups in 4-cumylphenol. The introduction of 4-cumylphenol can delay the infestation time of the soy meal and reduce the degree of infection, and control the growth of *Aspergillus* fungi inside the soy meal [19]. 

### 3.2. SEM Analysis of the Adhesives

**Figure 2:**
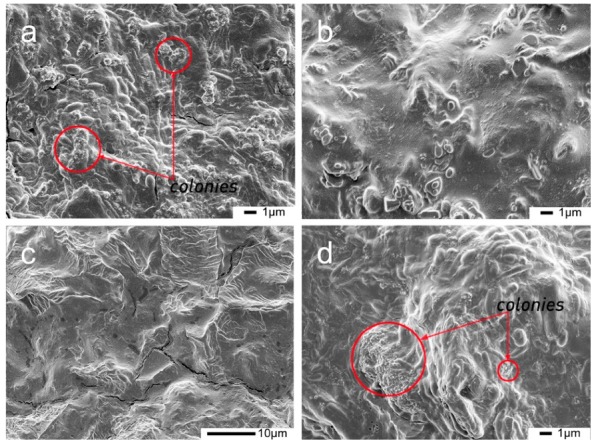
SEM of the different soy meal (SM) samples after 15 days of cultivation at 28 °C and 95% RH: (**a**) (SM/5% preservative A), (**b**) (SM/5% preservative B), (**c**) (SM/5% preservative C), and (**d**) (SM).

After being cultivated for 15 days, the whole area of samples K and A4 was densely filled with microorganisms (Figure 2a,d). However, no spores and mycelia were found in samples B4 and C4 (Figure 2b,c). The results indicate that the inhibitory effect of preservatives B and C did in fact exist in the adhesive samples, even if the microbial growth was solely found on the surfaces during visual observation (Figure 1).

Sample A4, which contained the preservative nano-Ag/TiO_2_, had poor mildew resistance. This phenomenon may result from the characteristics of TiO_2_. As is known, the active hydroxyl groups and superoxide ions produced by TiO_2_ under ultraviolet light can react with the cell walls, cell membranes, and intracellular components of bacteria or fungi to inhibit and kill bacteria [24]. However, nano-TiO_2_ has a large specific surface area and is easy to agglomerate. After agglomeration, the particles increase, which seriously affects the anti-mildew and antibacterial effect [26]. This also indirectly affects the display of the antimicrobial properties of the silver particles it supports. Researchers usually apply a coupling agent to overcome this flaw [35,36,37]. In addition, the antibacterial effect of nano-TiO_2_ is related to the light source, and its catalytic performance is the strongest under ultraviolet light conditions [27]. This experiment was carried out in a dark environment, which affected its catalytic performance and made the antibacterial effect not good.

Sample B4 contained the preservatives nano-Ag/TiO_2_ and ZPT. ZPT works as an antibacterial and antifungal agent and is widely used as a preservative in cosmetics and personal care products [31,37]. The main active component of nano-Ag/TiO_2_ is silver, which is a new broad-spectrum, highly effective antibacterial agent. When silver is in contact with bacteria, the unsaturated coordination ability of silver interacts with nitrogen or oxygen on the surface of bacteria or fungi, destroying the cell structure, allowing the bacterial contents to flow out, and acting as a bactericidal effect [22]. The synergistic effect produced by mixing nano-Ag/TiO_2_ and ZPT can further improve the anti-mildew and antibacterial properties. 

Phenolics are a major group of compounds that are considered to be toxic to microorganisms. They can potentially inactivate enzymes, intercalate into the cell wall or DNA, and disturb the function of bacterial cell membranes, which causes the retardation of both the growth and multiplication of bacteria [38]. The US Forest Products Laboratory conducted an experimental study on the anti-mildew and antibacterial properties of soy protein-based adhesives and their bonded sheets. Experiments show that chlorophenol, O-phenylphenol sodium salt, chlorophenol sodium salt, and O-phenylphenol are considered to be more effective soy protein-based adhesive antifungal and antibacterial agents [38,39]. The 4-cumylphenol compound has one phenolic hydroxyl group. The site and number of hydroxyl groups on the phenol group could possibly be responsible for the antimicrobial activities [18]. Hence, no fungal growth was observed on the surface of sample C4, which contained the preservative 4-cumylphenol after 15 days of cultivation.

### 3.3. Solid Content and Initial Viscosity

The solid content and initial viscosity are two important factors of soy-based adhesive in adhesive bonding strength [5]. These results showed that these preservative agents did not significantly change the SM characteristics for these two indicators, which is favorable for the utilization of modified SM as the raw material of soy-based adhesive (Table 3).

### 3.4. FTIR Analysis

**Figure 3:**
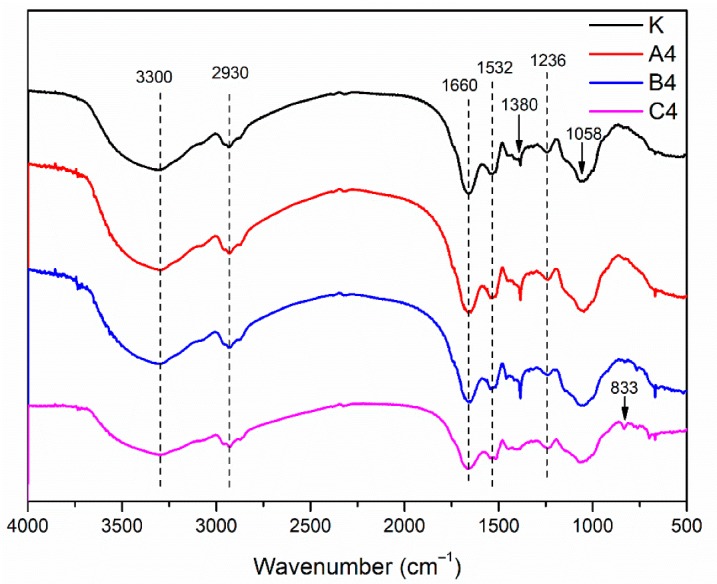
FTIR spectrum of the different new-made soy meal samples: K (SM adhesive), A4 (SM/5% preservative A), B4 (SM/5% preservative B), and C4 (SM/5% preservative C).

**Figure 4:**
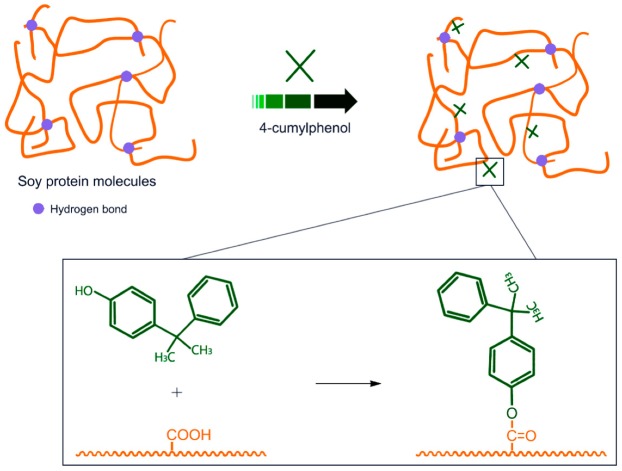
Interactions between soy protein and 4-cumylphenol.

In the spectra of different new-made samples (Figure 3), the absorption peaks were generally consistent, which indicates that the addition of different 5% preservatives (preservative A/preservative B) did not change the chemical construction of the soy meal. For these four groups of samples, the broad band observed in the range of 3500–3000 cm^−1^ was assigned to the free and bound O–H and N–H groups. As for the absorption bands of amide, the primary characteristic absorption bands appeared at 1660 cm^−1^ (amide Ⅰ), 1532 cm^−1^ (amide Ⅱ), and 1236 cm^−1^ (amide Ⅲ), which were assigned to C=O stretching, N–H bending, and C–N and N–H stretching, respectively [18]. The absorption peak at 2930 cm^−1^ was assigned to the symmetric and asymmetric stretching vibrations of the –CH_2_ groups in the SM [40,41,42]. The COO– and –C–NH_2_ absorptions were seen at 1380 and 1058 cm^−1^, respectively. The absorption peak at about 670 cm^−1^ were attributed to C–O–H out-of-plane bending in samples A4, B4, and C4. The main difference between the spectra of soy adhesive with or without 4-cumylphenol came from the absorptions at 833 cm^−1^, which were assigned to C–O–C stretching [16]. It may also be attributed to phenol–O–C in preservative 4-phenylphenol. The absorption at 1380 cm^−1^ became weaker than that in pure soy meal in sample C4, which might be a result of the reaction between the methylol group of 4-cumylphenol and the COO– groups of soy meal (Figure 4). 

### 3.5. Thermal Stability Measurements

**Figure 5:**
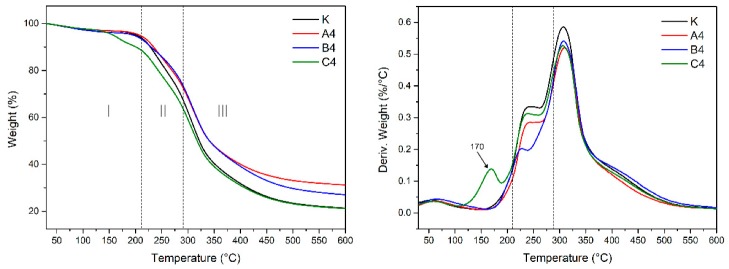
TG and differential TG curves of the different new-made soy meal samples: K (SM adhesive), A4 (SM/5% preservative A), B4 (SM/5% preservative B), and C4 (SM/5% preservative C).

The thermogravimetric curve of the pure soy meal (sample K) is shown in Figure 5. The thermal degradation of soy protein can be divided into three stages. The first stage of temperature up to 208 °C is the post-cure stage, in which the system is further cured, releasing moisture and gas, and there is mass loss. The second stage temperature is 247 °C, and a degradation peak appears around 240 °C. This stage mainly results from the degradation of some small molecules and unstable connections. The third stage belongs to the degradation stage of the skeleton structure, the temperature range is 283 to 327 °C, and a significant degradation peak appears at 304 °C. The degradation at this stage is mainly due to intermolecular and intramolecular hydrogen bonding, electrostatic binding, and cleavage of the soy protein molecular chain itself.

The comparison of the thermogravimetric curves of the blank sample and the soy meal with the three anti-mold agents added is shown in Figure 5. It can be seen from the comparison chart that a new peak was observed at 170 °C in the curve C4, indicating a different structure formed in the cured system after adding 4-cumylphenol as a preservative agent. The new structure was formed by the reaction between 4-cumylphenol and protein molecules according to the analysis of the FTIR. Second, the thermal degradation behavior was nearly consistent between the four samples at stages II and III. 

**Figure 6:**
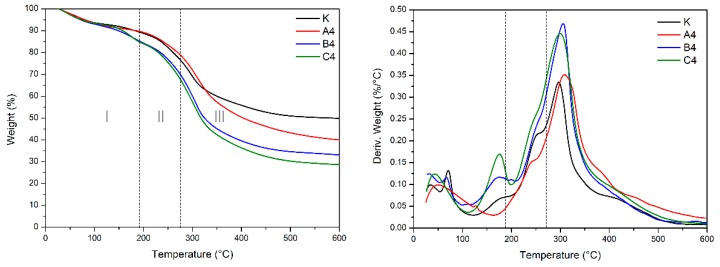
TG and differential TG curves of the different soy meal samples on the 15th day: K (SM adhesive), A4 (SM/5% preservative A), B4 (SM/5% preservative B), and C4 (SM/5% preservative C).

The thermogravimetric curve after 15 days of mildew observation of the blank sample and the soy meal with the three anti-mold agents added is shown in Figure 6. The remaining weight percentage of sample K on the 15th day was up to 50%, which was caused by a large amount of ash produced by mold growth and metabolism. Comparing with Figure 5, the curves of samples B4 and C4 did not changed too much, which means the composition of samples B4 and C4 has almost unchanged, indicating that the mold inhibitors B and C inhibit the degradation of the soybean protein by the microorganisms to some extent. 

## 4. Conclusions

To improve the anti-mold capacity of soy meal, three kinds of preservatives, namely nano-Ag/TiO_2_, nano-Ag/TiO_2_ with ZPT, and 4-cumylphenol were used in this study. Both thermogravimetry results and surface morphology analysis indicated that nano-Ag/TiO_2_ with ZPT and 4-cumylphenol improved the anti-mold capacity of the soy-based adhesive. Nano-Ag/TiO_2_ had a poor anti-mildew effect on soy meal. However, with an increase in additive concentration, the anti-mildew ability of the soy meal continued to increase. Based on the results of FTIR analysis, the chemical construction of soy meal with nano-Ag/TiO2 and nano-Ag/TiO_2_ with ZPT did not change. When the amount of the preservative nano-Ag/TiO_2_ with ZPT was 0.5 wt %, no mold was seen on the surface by visual observation after 15 days of culturing. Meanwhile, the amount of the preservative 4-cumylphenol needed to be 2 wt % in order to achieve the same effect. The results were attributed to the following: (i) the reaction between the methylol group of 4-cumylphenol and COO– groups of soy meal; (ii) the soy meal modified using nano-Ag/TiO_2_ with ZPT and 4-cumylphenol could effectively resist the fungi and mold, contributing to the industrialization of SM-based adhesives.

## Figures and Tables

**Table 1:** Experimental formulation details for soy meal samples.

Sample Number	Type of Preservative	Amount of Preservative(g)
K	—	—
A1	Nano-Ag/TiO2	0.5
A2	Nano-Ag/TiO2	1
A3	Nano-Ag/TiO2	2
A4	Nano-Ag/TiO2	3
B1	Nano-Ag/TiO2 & ZPT	0.5
B2	Nano-Ag/TiO2 & ZPT	1
B3	Nano-Ag/TiO2 & ZPT	2
B4	Nano-Ag/TiO2 & ZPT	3
C1	4-Cumylphenol	0.5
C2	4-Cumylphenol	1
C3	4-Cumylphenol	2
C4	4-Cumylphenol	3

The mass ratio of Nano-Ag/TiO2 & ZPT is 1:1.

**Table 2:** Degree of mold growth on the surface of samples at 28 °C and 95% RH for 15 days.

Time (Day)	Degree of Mold Growth												
	K	A1	A2	A3	A4	B1	B2	B3	B4	C1	C2	C3	C4
**1**	**+**												
**2**	**++**	**++**	**++**	**+**						**+**			
**3**	**+++**	**+++**	**+++**	**+++**						**+**			
**4**	**++++**	**++++**	**++++**	**++++**	**+**					**+**	**+**		
**5**	**+++++**	**+++++**	**+++++**	**+++++**	**+**					**++**	**+**		
**6**	**+++++**	**+++++**	**+++++**	**+++++**	**++**					**++**	**+**		
**7**	**+++++**	**+++++**	**+++++**	**+++++**	**+++**					**+++**	**+**		
**8**	**+++++**	**+++++**	**+++++**	**+++++**	**++++**					**++++**	**+**		
**9**	**++++++**	**++++++**	**++++++**	**++++++**	**++++**					**+++++**	**+**		
**10**	**++++++**	**++++++**	**++++++**	**++++++**	**+++++**					**++++++**	**+**		
**11**	**++++++**	**++++++**	**++++++**	**++++++**	**+++++**					**++++++**	**+**		
**12**	**++++++**	**++++++**	**++++++**	**++++++**	**+++++**					**++++++**	**+**		
**13**	**++++++**	**++++++**	**++++++**	**++++++**	**+++++**					**++++++**	**+**		
**14**	**++++++**	**++++++**	**++++++**	**++++++**	**++++++**					**++++++**	**+**		
**15**	**++++++**	**++++++**	**++++++**	**++++++**	**++++++**					**++++++**	**+**		

Blank stands for absence of growth. + infected area < 25%, ++ 25% < infected area < 50%, +++ 50% < infected area < 75%, ++++ 75% < infected area < 100%, +++++ 100% of adhesive surface was covered by microorganisms. ++++++ Change in mycelium color.

**Table 3:** Solid content and initial viscosity.

	Adhesive Code												
	K	A1	A2	A3	A4	B1	B2	B3	B4	C1	C2	C3	C4
Solid content(%)	26.77	26.91	27.15	27.32	27.46	27.12	27.42	27.68	27.83	26.83	26.85	27.45	27.61
Initial viscosity (mPa·s)	38,432	31,063	30,913	30,471	30,226	49,697	50,149	50,685	51,092	34,193	37,152	37,639	38,273
